# Supplementary material for: Moderate Weight Loss Modifies Leptin and Ghrelin Synthesis Rhythms but Not the Subjective Sensations of Appetite in Obesity Patients
Source: Nutrients. 2020 Mar 27;12(4):916. doi: 10.3390/nu12040916 (PMC7230904; doi:10.3390/nu12040916)
Supplement: Supplementary file 1 [file nutrients-12-00916-s001.pdf]

Table S1. Energy and nutrient composition of the test meal supplied to participants.

**Breakfast**	Calories /portion	CARBOHYDRATES(g) /portion	Sugars (g) /portion	PROTEINS(g) /portion	FAT(g) /portion	SATURATED FAT(g) /portion	SALT(g) /portion	FIBER(g) /portion
Skimmed Milk (175 ml) [Central Lechera Asturiana]	84,00	14,80	14,80	5,60	0,13	0,09	0,09	0,00
Coffee(8g) [Saimaza]	18,20	3,30		1,20	0,04	0,02		0,00
Sugar (4g) [Azucarera Española]	16,00	4,00		0,00	0,00	0,00		0,00
Banana (105g) [Hacendado]	81,50	17,80		0,91	0,23	0,10		2,20
Cookies (80g; 40g/unidad) [All-Bran]	306,00	46,00	30,00	3,00	10,00	0,80	0,36	7,00
	505,70	85,90	44,80	10,71	10,40	1,00	0,45	9,20

**Lunch**	Calories /portion	CARBOHYDRATES(g) /portion	Sugars (g) /portion	PROTEINS(g) /portion	FAT(g) /portion	SATURATED FAT(g)/portion	SALT(g) /portion	FIBER(g) /portion
Sandwich (130g) [Campofrío]	301,00	34	1,9	10	10	3	1,8	0,00
Potato Chips (30g) [Hacendado]	165	15,8	0,22	1,83	10,8	1,35	0,3	0,53
Tangerines (120g) [Hacendado]	20,4	4,2		0,29	0,091	0,01		0,82
Nuts (30g) [Hacendado]	195	1,3		4,3	18,8	2		1,7
Chocolate Snack (Twix) (50g)	124	16,1	12,2	1,1	6	3,5	0,11	
	805,4	71,4	14,32	17,52	45,69	9,86	2,21	3,05

**Dinner**	Calories /portion	CARBOHYDRATES(g) /portion	Sugars (g) /portion	PROTEINS(g) /portion	FAT(g) /portion	SATURATED FAT(g)/portion	SALT(g) /portion	FIBER(g) /portion
Sandwich (130g) [Campofrío]	301	34	1,9	10	10	3	1,8	0,00
Potato Chips (30g) [Hacendado]	165	15,8	0,22	1,83	10,8	1,35	0,3	0,53
Tangerines (120g) [Hacendado]	20,4	4,2		0,29	0,091	0,01		0,82
Nuts (30g) [Hacendado]	195	1,3		4,3	18,8	2		1,7
Apple/cereal Snack [Hero] (30g)	74	13	5,6	1,3	1,4	0,7		2,6
	755,4	68,3	7,72	17,72	41,09	7,06	2,1	5,65

Table S2. Summary of energy and nutrient composition of the test meal.

	**Total Nutrient Composition**
**Weight (g)**	1072
**Kilocalories**	2067,0
**Kjul**	8654.12
**Carbohydrates (g)**	225.60
**% Energy from Carbohydrates**	50
**Sugars (g)**	66.84
**Proteins (g)**	45.95
**% Energy from Proteins**	10

**Fats** (g)	97.18
**% Energy from Fats**	40
**Saturated Fat** (g)	17.92
**Fiber** (g)	17.90
**Salt** (g)	4.76

Table S3. Significant correlation coefficients between the data derived from the rhythmic analysis (cosinor method) and the indicators of diet effectiveness

	**Body weight (Δkg)**		**BMI (Δkg/m²)**		**Body fat (Δ%)**		**waist circumference (Δcm)**	
**Subjects losing >10% body weight (n=18)**	*r*	*p*	*r*	*p*	*r*	*p*	*r*	*p*
**Leptin**								
Mesor	0.478	0.045	-	-	-	-	-	-
Acrophase	-	-	-	-	-	-	-0.502	0.034
%Rhymicity	-	-	-	-	-	-	-	-
**Ghrelin**								
Mesor	-	-	-	-	-	-	-	-
Acrophase	-	-	-	-	-	-	-	-
%Rhymicity	-	-	-	-	-	-	0.482	0.043
**Glucose**								
Mesor	-0.607	0.008	-0.572	0.013	-	-	-	-
Acrophase	-	-	-	-	-	-	-	-
%Rhymicity	-	-	-	-	-	-	-	-
**Hunger**								
Mesor	-	-	-	-	-	-	-	-
Acrophase	-	-	-	-	-	-	-0.639	0.004
%Rhymicity	-	-	-	-	-	-	-	-
**Satiety**								
Mesor	-	-	-	-	-	-	-	-

Acrophase	-	-	-	-	-0.476	0.046	-	-
%Rhymicity	-	-	-	-	-	-	-	-
**Desire to Eat**								
Mesor	-	-	-	-	-	-	-	-
Acrophase	-	-	-	-	-	-	-0.513	0.030
%Rhymicity	-	-	-	-	0.468	0.050	-	-
